# Chromatin roadblocks to reprogramming 50 years on

**DOI:** 10.1186/1741-7007-10-83

**Published:** 2012-10-29

**Authors:** Peter J Skene, Steven Henikoff

**Affiliations:** 1 Howard Hughes Medical Institute, Fred Hutchinson Cancer Research Center, Seattle, Washington 98109, USA

## Abstract

A half century after John Gurdon demonstrated nuclear reprogramming, for which he was awarded the 2012 Nobel Prize in Physiology or Medicine, his group provides insights into the molecular mechanisms whereby chromatin remodeling is required for nuclear reprogramming. Among the issues addressed in Gurdon's latest work are the chromatin impediments to artificially induced reprogramming, discovered by Shinya Yamanaka, who shared the award with Gurdon.

See research article: http://www.epigeneticsandchromatin.com/content/5/1/17

## 

Nuclear reprogramming is the name given to the change that occurs in the nucleus of a somatic cell when it is induced to revert from its differentiated state and assumes a pluripotent state, from which it can adopt any cellular identity, given the appropriate cues. This is the basis for much of the promise in regenerative medicine, for example in the repopulation of bone marrow following chemotherapy. In a laboratory setting, reprogramming can be achieved in two ways. In the first, the somatic cell nucleus is transferred into an oocyte. Here the oocyte provides the necessary factors to reprogram the somatic nucleus, which is in principle then capable of recapitulating the entire developmental program. In the second, expression of four key transcription factors (Oct4, Sox2, Klf4 and c-Myc) is sufficient to reprogram a somatic cell to produce what is known as an induced pluripotent stem cell (iPSC) state, originally discovered by Shinya Yamanaka and colleagues [1].

There are, however, several hurdles to be overcome before reprogrammed cells can be used in a therapeutic setting. Currently, the generation of iPSCs is typically slow and the reprogramming of somatic cells from accessible adult tissues, which is most applicable for therapeutic uses, is particularly inefficient because donor cells from these tissues are at a late stage of differentiation [2]. It also appears that iPSCs are not truly equivalent to the pluripotent embryonic stem cell, as iPSCs display a reduced differentiation capacity that is biased to the cell lineage of origin. This is consistent with the observation that iPSCs may retain a memory of the somatic cell gene expression pattern. An important aim of research in this field, therefore, is a better understanding of the mechanism of reprogramming that may lead to improvements in the efficiency and fidelity with which pluripotent stems cells can be generated. Such an understanding is beginning to emerge from studies on chromatin remodeling in the generation of pluripotent stem cells.

## Resetting the chromatin landscape

Studies on iPSC generation have suggested that chromatin at the promoters and enhancers of pluripotency genes in somatic cells is in a repressed state characterized by modifications such as DNA methylation and histone deacetylation, and this is a roadblock to reprogramming, which is thus promoted by inhibitors of DNA methylation and histone deacetylation [3]. c-Myc is thought to function as a catalyst in this process, by increasing the rate of cell proliferation and perhaps transcriptional elongation, both of which result in large-scale chromatin remodeling.

In the study reported in *Epigenetics and Chromatin*, John Gurdon and colleagues describe the investigation of reprogramming that is independent of DNA replication, by transferring mammalian somatic cell nuclei into *Xenopus *oocytes, which are mitotically arrested, and following the resulting chromatin changes [4]. They focused on the incorporation of the histone variant H3.3, which is a hallmark of sites of high nucleosome turnover, and is associated with active genes and their regulatory elements [5,6]. When they microinjected mRNA encoding epitope-tagged H3.3 into the oocytes prior to nuclear transfer, they observed early incorporation of H3.3 into the pluripotency gene *Oct4 *coincident with the onset of transcription of the gene. To check the requirement for H3.3, they injected into the oocyte polyclonal antibodies against HIRA, the chaperone responsible for the incorporation of histone H3.3 into chromatin, and were able to show that this abrogates transcriptional reprogramming. They also showed, by the use of the polymerase II inhibitor alpha-amanitin, that H3.3 incorporation depends on transcriptional activity as well as HIRA. Impaired reprogramming in the absence of HIRA and H3.3 deposition could not be compensated for by the increased deposition of histone variant H3.2. These results imply that some specific function is attributable to the H3.3 deposition pathway in promoting reprogramming, and raises the question of what that function might be.

Reprogramming is far less efficient than differentiation and this may reflect the need for reprogramming factors to overcome changes to the chromatin environment that occur with differentiation. Embryonic stem cells are characterized by a highly dynamic chromatin state compared with that of more differentiated cell types [7], and pluripotency genes in particular have been shown to gain repressive chromatin marks during differentiation. It is, however, at these silenced sites that the reprogramming factors must bind to elicit their effects. Whilst c-Myc binding occurs early in the reprogramming process, Oct4, Sox2 and to a lesser extent Klf4, which co-occupy a large number of promoters, bind only later during reprogramming. Delayed binding of Oct4, Sox2 and Klf4, presumably because of the repressive chromatin environment at their binding sites, is thought to be a major roadblock in the reprogramming process.

As transcription factor binding sites associated with active genes are marked by rapidly turning-over nucleosomes, it is probable that during reprogramming the nucleosomes at the Oct4/Sox2/Klf4 sites become remodeled to contain H3.3. The high turnover observed at regulatory sites might be required to prevent sites from becoming stably occupied by nucleosomes, which would impede transcription factor binding and therefore reprogramming. This removal of repressive chromatin and replacement with H3.3 may facilitate transcription factor binding and successful reprogramming. Perhaps, therefore, HIRA-dependent H3.3 deposition is functioning to promote pluripotency. This is consistent with the observation that HIRA deficient embryonic stem cells rapidly differentiate [7]. It also, however, raises the question of how histone H3.3 may confer a more accessible chromatin environment.

## Histone H3.3: striking a balance

One obvious possibility is that the high turnover of H3.3-containing nucleosomes reflects some intrinsic instability. However, H2A/H3.3-containing nucleosomes seem to be just as stable *in vitro *as H2A/H3.1-containing nucleosomes [8]. Another possibility is that histone H3.3 accumulates at these high turnover sites as a gap-filling mechanism after the eviction of nucleosomes following transcription or the activity of chromatin remodelers. The observations of Gurdon and colleagues, however, indicate failure of histone variant H3.2 to compensate for the lack of H3.3 deposition, suggesting that there is more to H3.3 deposition in the reprogramming process than simple gap filling.

**Figure 1:**
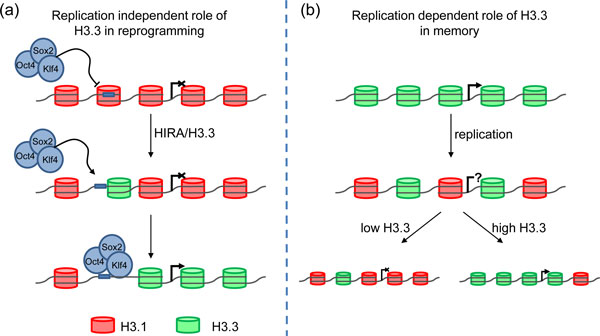
**Opposing roles of histone H3.3 in reprogramming**. **(a) **In somatic cells, pluripotency genes are in a repressive chromatin environment. Transient binding by a reprogramming factor to its binding site results in histone H3.3 incorporation, which in turn results in a more permissive chromatin structure and enhanced accessibility of the binding site. These chromatin changes increase the probability of concomitant binding of Oct4, Sox2 and Klf4 and activation of the target pluripotency gene. **(b) **Active genes are marked by high levels of histone H3.3. During DNA replication H3.3 is diluted out by the replication-dependent incorporation of histone H3.1. Depending on appropriate cues the gene will either become re-activated or become silenced. During reprogramming, tissue-specific genes become progressively silenced. High levels of H3.3 expression, however, increase the probability of H3.3 incorporation and therefore promote the memory of the somatic cell gene expression pattern.

If there is nothing intrinsic to the amino acid sequence of H3.3 that favors a permissive chromatin state, then perhaps the deposition process itself is key. Perhaps, therefore, a specific post-translational modification of H3.3 might be associated with HIRA-dependent deposition, or an additional chromatin remodeling step at the point of H3.3 deposition might promote an active chromatin conformation and accessibility to the underlying DNA sequence. iPSC generation has been suggested to be a stochastic process [9], which at a molecular level may reflect the stochastic nature of binding by the reprogramming factors to their target sequences due to transient exposure of these sites in the repressive chromatin environment [10]. Increases in the deposition of H3.3 may therefore facilitate reprogramming by increasing the frequency of exposure of these binding sites (Figure 1a).

A key question for future studies is whether the overexpression of HIRA and/or histone H3.3 may accelerate reprogramming and increase the efficiency of iPSC generation by allowing the remodeling of transcription factor binding sites. A second, related question is whether such overexpression might provide the necessary replication-independent chromatin remodeling, thereby reducing the requirement for c-Myc as a catalyst. This would be desirable because c-Myc, which is thought to aid reprogramming by increasing the rate of cell proliferation and thereby genome-wide chromatin remodeling, is a proto-oncogene; there are thus naturally concerns about its induced expression in the generation of iPSCs as a therapeutic agent.

There is, however, a potential pitfall in the overexpression of HIRA/H3.3 in reprogramming. Whilst it may allow the remodeling of repressive chromatin to promote gene activation, histone H3.3 has also been shown in an earlier study by Gurdon and colleagues [11] to potentiate transcriptional memory (Figure 1b). In that study, using reprogramming via somatic cell nuclear transfer into enucleated *Xenopus *oocytes, they showed that overexpression of histone H3.3 increased the frequency at which nuclei maintained their original transcriptional program, as determined by *MyoD *expression. They have since suggested that the unusually high H3.3 content in eggs is responsible for this transcriptional memory [2]. This may suggest the need for a delicate balance in the overexpression of HIRA and/or H3.3, in which factor binding will be facilitated through chromatin decondensation, but transcriptional memory will not be evoked. Given the evidence that transcriptional memory in iPSCs may cause some of the observed limitations to the regenerative applications of these cells, clearly the operation of the H3.3 pathway in both reprogramming and memory will need to be taken into account in any manipulation of that pathway for therapeutic purposes. Gurdon's latest work thus forges a connection, through the changes to chromatin required for reprogramming, between the two 2012 Nobel Prize-winning papers published almost a half century apart.
